# The clinical relevance of laboratory prognostic scores for patients with radiosurgically treated brain metastases of non-pulmonary primary tumor

**DOI:** 10.1007/s11060-021-03788-6

**Published:** 2021-06-20

**Authors:** Anna Cho, Helena Untersteiner, Fabian Fitschek, Farjad Khalaveh, Philip Pruckner, Noemi Pavo, Karl Rössler, Christian Dorfer, Brigitte Gatterbauer, Christoph Höller, Manuela Schmidinger, Josa M. Frischer

**Affiliations:** 1 Department of Neurosurgery, Medical University of Vienna, Waehringer Guertel 18-20, 1090 Vienna, Austria; 2 Department of General Surgery, Medical University of Vienna, Vienna, Austria; 3 Department of Internal Medicine II, Division of Cardiology, Medical University of Vienna, Vienna, Austria; 4 Department of Dermatology, Medical University of Vienna, Vienna, Austria; 5 Department of Urology, Medical University of Vienna, Vienna, Austria

**Keywords:** Gamma Knife Radiosurgery, Brain metastases, Prognostic scores, NLR, mGPS

## Abstract

**Purpose:**

To investigate the clinical value of the inflammation based prognostic scores for patients with radiosurgically treated brain metastases (BM) originating from non-pulmonary primary tumor (PT).

**Methods:**

A retrospective analysis of 340 BM patients of different PT origin (melanoma, breast, gastrointestinal, or genitourinary cancer) was performed. Pre-radiosurgical laboratory prognostic scores, such as the Neutrophil-to-Lymphocyte Ratio (NLR), the Platelet-to-Lymphocyte Ratio (PLR), Lymphocyte-to-Monocyte Ratio (LMR), and the modified Glasgow Prognostic Score (mGPS), were investigated within 14 days before the first Gamma Knife radiosurgical treatment (GKRS1).

**Results:**

In our study cohort, the estimated survival was significantly longer in patients with NLR < 5 (p < 0.001), LMR > 4 (p = 0.001) and in patients with a mGPS score of 0 (p < 0.001). Furthermore, univariate and multivariate Cox regression models revealed NLR ≥ 5, LMR < 4 and mGPS score ≥ 1 as independent prognostic factors for an increased risk of death even after adjusting for age, sex, KPS, extracranial metastases status, presence of neurological symptoms and treatment with immunotherapy (IT) or targeted therapy (TT).

**Conclusions:**

Summarizing previously published and present data, pre-radiosurgical mGPS and NLR groups seem to be the most effective and simple independent prognostic factors to predict clinical outcome in radiosurgically treated BM patients.

## Introduction

Over the last decades, there has been an increase in the incidence of brain metastses (BM) due to improvements in diagnostic workups, but also in systemic oncological therapies [1]. Although lung cancer represents the most common primary tumor (PT), the incidence of BM from other PT, such as melanoma, breast, renal, colorectal cancer should not be underestimated [1, 2].

In BM patients, several prognostic factors, including the PT histology, patient’s age, the systemic disease status, the Karnofsky Performance Status (KPS), as well as the number, size, and localization of BM, are of utmost interest [2]. Recently, several laboratory prognostic predictors for solid and metastatic cancer patients have additionally gained interest due to their simplicity and wide availability [3–5]. Therefore, leucocyte-based ratios, such as the Neutrophil-to-Lymphocyte Ratio (NLR), the Platelet-to-Lymphocyte Ratio (PLR), and the Lymphocyte-to-Monocyte Ratio (LMR), have been previously reported to be prognostic for overall survival in patients with surgically treated BM [4, 6]. However, the clinical data on NLR, PLR and LMR for radiosurgically treated BM patients are still mainly limited to non-small cell lung cancer (NSCLC) patients [7–9].

Moreover, the modified Glasgow Prognostic Score (mGPS), consisting of albumin and C-reactive protein (CRP), has also shown to be prognostic for survival in cancer patients [10–12]. However, mGPS has rather been applied for solid cancers so far, and clinical data in radiosurgically treated BM patients are still lacking.

The aim of the study was to investigate the clinical value of these laboratory prognostic scores in radiosurgically treated BM patients of non-pulmonary PT origin.

## Materials and methods

For this retrospective study, patients who have been treated between June 2012 (implementation of the new Gamma Knife® Perfexion™ at our institution) and December 2019 were included. Our inclusion criteria were patients with an age > 18 years, at least one Gamma Knife radiosurgical (GKRS) treatment for at least one BM from melanoma, breast cancer, gastrointestinal (GI) or genitourinary (GU) cancer, and available laboratory parameters within 14 days before the first GKRS (GKRS1) [7].

**Fig. 1:**
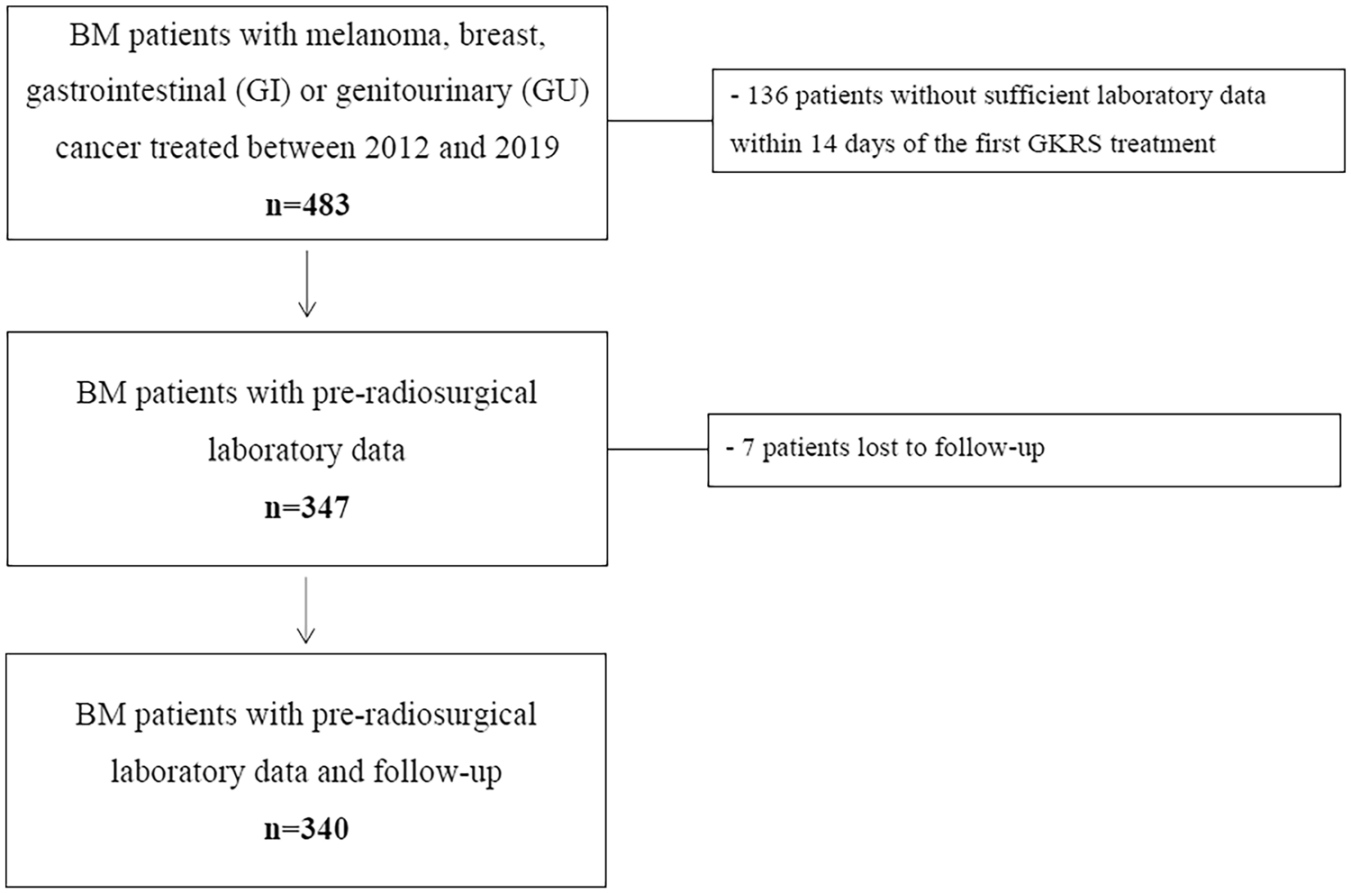
**Flow chart depicting the study inclusion algorithm**. This figure gives an overview of our study patient selection. At our institution, 483 patients with BM from melanoma, breast cancer, GI or GU cancer were treated with GKRS for at least one BM between June 2012 (implementation of the new Gamma Knife® Perfexion™ at our institution) and December 2019. After excluding 136/483 (28%) patients without archived or sufficient laboratory parameters within 14 days before GKRS1, and 7/483 (1%) patients with lost to follow-up, a total of 340/483 (71%) patients could be enrolled in this study. *BM* brain metastases, *GKRS* Gamma Knife Radiosurgery, *GKRS1* first Gamma Knife radiosurgical treatment, *GI* gastrointestinal, *GU* genitourinary

After excluding patients according to our criteria, and patients lost to follow-up (FU), 340 patients could be enrolled in this study (Fig. 1).

Subsequently, all patients with available laboratory findings for the calculation of NLR, PLR, and LMR, were classified according to the previously defined cut-off-values. The cut-off values were 5 for NLR, 180 for PLR and 4 for LMR [4, 7, 13]. As previously reported, the mGPS was defined as follows: no abnormalities in CRP (≤ 10 mg/l) and albumin (≥ 35 g/l) was classified as a mGPS score of 0, while both abnormalities were classified as a mGPS score of 2. For patients with elevated CRP but without decreased albumin, a mGPS of 1 was allocated [14].

To review the clinical value of prognostic scores in relation to concomitant modern oncological therapies, data on immunotherapy (IT) or targeted therapy (TT) were additionally reviewed at the time of GKRS1 (± 30 days). The study complied with the Declaration of Helsinki and was approved by the local ethics review committee (EK1788/2020).

### Radiosurgical technique

As we have previously described, radiosurgical treatments were planned with GammaPlan and performed with Leksell Gamma Knife® Perfexion™ (Elekta AB, Stockholm, Sweden). The planning sequences were performed on a 1.5 or 3.0 Tesla magnet MRI and always included Gadolinium contrast-enhanced T1-weighted MRI sequences in axial and coronal planes [15].

The median time between initial BM diagnosis and GKRS1 was 0.5 months (0.0–99.4). About two-thirds of the study population (204/340, 60%) underwent only one GKRS treatment, while the others (136/340, 40%) received multiple treatments due to newly diagnosed BM or two-fraction dose-staged GKRS, as described before [16].

The median treatment volume was 0.6 cm^3^ (0.1–29.0). The prescribed doses mainly targeted the 50% (45–90) isodose line, with a median prescription dose of 18 Gy (6–24) and a median central dose of 32 Gy (12–45).

### Outcome evaluation and statistical analyses

According to our standard follow-up procedure, all radiosurgically treated patients were clinically and radiologically assessed in a 3-month interval. The primary study endpoint was defined as all-cause mortality. For further outcome data evaluation, a death register comparison was performed.

Categorical data were presented as counts and percentages, and continuous parameters as median and range. The chi-square, Mann–Whitney U, and Wilcoxon signed-rank tests were performed as statistically appropriate.

Median survival after the first GKRS was estimated by the Kaplan–Meier method and compared with the Log-Rank-Test. Univariate and multivariate Cox proportional hazard regression analyses were performed to estimate the association of the prognostic scores with mortality and to evaluate for potential confounding clinical parameters. Multivariate Cox regression analyses included sex, age group (≤ 65 vs. > 65a), KPS group (< 80% vs. ≥ 80%), the presence of extracranial metastases (ECM), the presence of neurological symptoms (asymptomatic versus symptomatic patients), the NLR, PLR, LMR, and mGPS groups [7].

For all tests, p values < 0.05 were considered to be statistically significant. Statistical analyses were carried out with IBM SPSS Statistics for Windows (Version 26.0 Armonk, NY: IBM Corp.).

## Results

### Patient characteristics and overall survival

The baseline characteristics of our study population are displayed in Table 1. The median age of the study population was 63 (29–89) years and 52% (178/340) of the patients were female. The most common PT in our study population was melanoma (149/340, 44%), followed by breast cancer (94/340, 28%), GI (55/340, 16%), and GU (42/340, 12%). The median time between the diagnosis of the PT and BM was 26.5 months (0.0–354.8). Furthermore, the estimated median survival time was 56.0 months [95% confidence interval (CI) = 46.3–65.8] after the initial diagnosis of the PT, 12.0 months (95% CI = 9.6–14.3) after the initial diagnosis of BMs, and 9.1 months (95% CI = 7.1–11.1) after GKRS1.

**Table 1:** Baseline characteristics of the study population

	Time of first GKRS (n = 340)
Age, in years, median (range)	60 (29–89)
Age groups	
≤ 65	201 (59%)
> 65	139 (41%)
Female: male ratio	178:162
KPS, in %, median (range)	80 (40–100)
KPS groups	
≥ 80%	243 (72%)
< 80%	97 (28%)
Neurological symptoms	
Yes	228 (67%)
No	112 (33%)
Primary tumor	
Melanoma	149 (44%)
Breast	94 (28%)
Gastrointestinal	55 (16%)
Genitourinary	42 (12%)
ECM Status at time of BM diagnosis	
Yes	302 (89%)
No	38 (11%)
IT and/or TT	
Yes	182 (53%)
No	152 (45%)
Unknown	6 (2%)
CNS treatment before GKRS1	
None	260 (76%)
WBRT and/or fRT	23 (7%)
BM resection without RT	34 (10%)
BM resection with WBRT and/or fRT	23 (7%)
Localization of BM at initial diagnosis	
Multiple	206 (61%)
Frontal	28 (8%)
Parietal	25 (7%)
Temporal	12 (4%)
Occipital	17 (5%)
Central	18 (5%)
Basal ganglia/brainstem/other	8 (2%)
Cerebellar	26 (8%)
Predicted survival after prognostic scores, in months, median (range)	
GPA general	3.8 (2.6–11.0)
GPA specific	7.7 (3.0–25.3)
RPA	4.5 (2.3–7.7)
SIR	6.0 (2.1–8.8)

This Table shows the detailed baseline characteristics of our 340 radiosurgically treated BM patients. Prior CNS treatment was mainly performed for distant BM. To review the clinical value of these prognostic scores in relation to IT or TT were reviewd at the time of the first GKRS treatment (± 30 days)

*BM* brain metastases, *CNS* central nervous system, *ECM* extracranial metastases, *fRT* fractionated radiotherapy, *GKRS* Gamma Knife Radiosurgery, *GPA* Graded Prognostic Assessment, *IQR* InterQuartile Range, *IT* immunotherapy, *KPS* Karnofsky Performance Status Scale, *RPA* Recursive Partitioning Analysis, *SIR* Score Index for Radiosurgery, *TT* targeted therapy, *WBRT* whole brain radiation therapy

The estimated median survival was significantly longer in female patients (p = 0.026), younger patients with an age of ≤ 65 years (p = 0.015), KPS of 80% or above (p < 0.001), and neurologically asymptomatic patients (p = 0.002). Interestingly, the presence of ECM was not significantly associated with shorter survival (p = 0.246).

After excluding six patients (6/340, 2%) without any known data on IT or TT, the survival after GKRS1 was evaluated for patients with and without IT or TT. This sub-analysis showed a significantly longer estimated survival in patients with IT or TT (182/340, 54%) than in patients without IT or TT (152/340, 44%; p = 0.036).

### Neutrophil-to-lymphocyte ratio

**Fig. 2:**
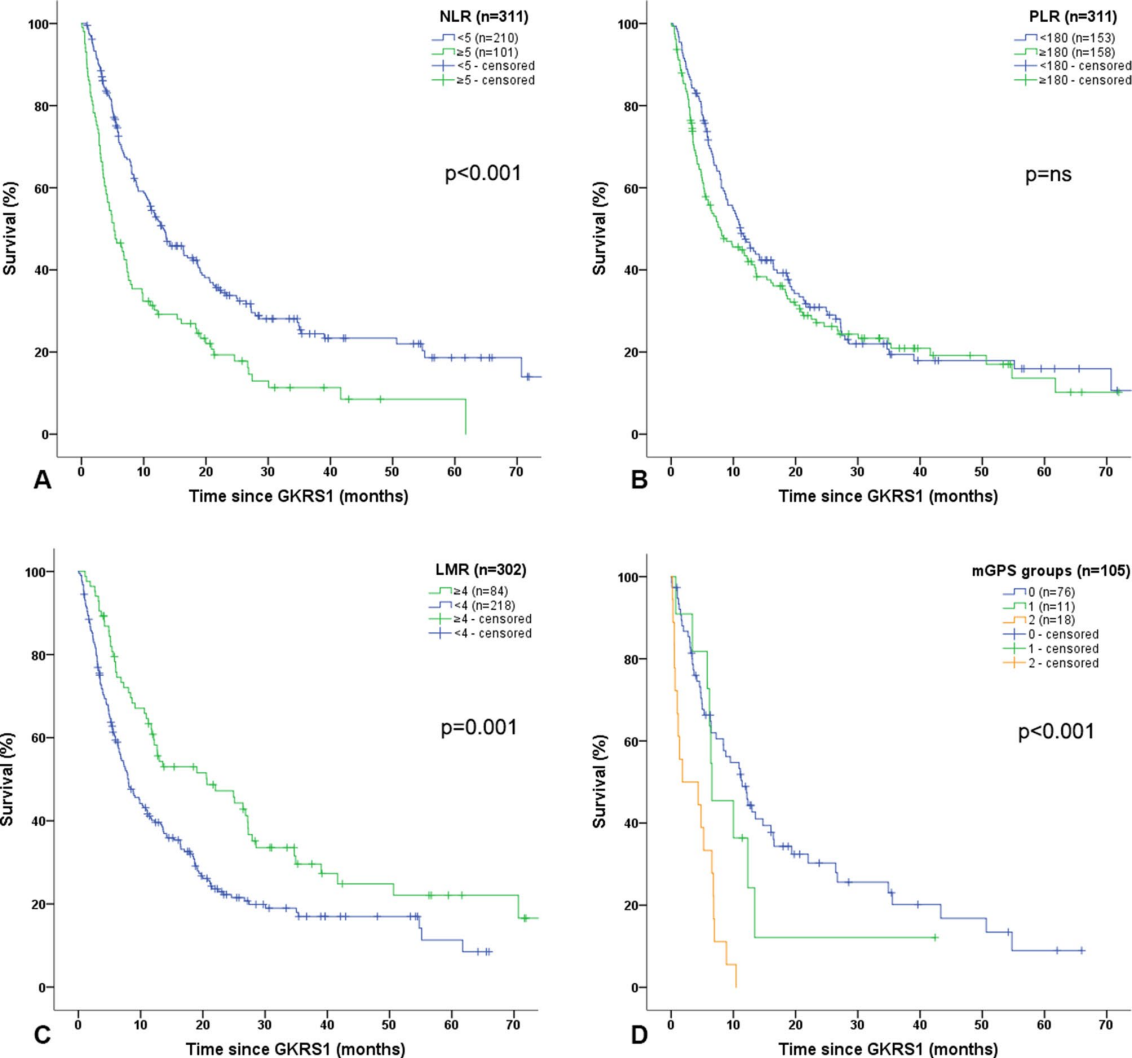
**Prognostic scores in relation to survival after GKRS1**. **A** *NLR in relation to survival after GKRS1*. In our study cohort, NLR values could be evaluated for 311/340 (92%) radiosurgically treated BM patients. Patients with NLR < 5 had a significant longer estimated median survival after GKRS1 (210/311, 68%; 13.2 months, 95% CI = 9.6–16.8) than those patients with NLR ≥ 5 (101/311, 32%; 5.2 months, 95% = CI 3.3–7.1; p < 0.001). **B** *PLR in relation to survival after GKRS1*. The estimated median survival did not show any significant differences between patients with PLR < 180 (153/311, 49%; 11.2 months, 95% CI = 8.6–13.7) and patients with PLR ≥ 180 (158/311, 51%; 7.9 months, 95% CI = 4.2–11.6; p = 0.226; Fig. 2B). **C** *LMR in relation to survival after GKRS1*. Due to missing monocyte values, the LMR values could be evaluated in 302/340 (89%) radiosurgically treated BM patients. The estimated median survival was significantly longer in patients with LMR ≥ 4 (84/302, 28%; 20.6 months, 95% = CI 8.6–32.7) than in patients with LMR < 4 (218/302, 72%; 8.0 months, 95% CI = 6.2–9.8; p = 0.001). **D** *mGPS in relation to survival after GKRS1*. Due to missing albumin values, mGPS could only be evaluated in 105/340 (31%) radiosurgically treated BM patients. The estimated median survival after GKRS1 was significantly longer in patients with a mGPS score of 0 (76/105, 72%; 11.4 months, 95% CI = 8.0–14.8) than in patients with mGPS score of 1 (11/105, 11%; 6.5 months, 95% CI = 2.4–10.7) or mGPS score of 2 (18/105, 17%; 1.8 months, 95% CI = 0.0–8.1; p < 0.001). *BM* brain metastases, *CI* confidence interval, *GKRS* Gamma Knife Radiosurgery, *GKRS1* first Gamma Knife radiosurgical treatment, *LMR* Lymphocyte-to-Monocyte-Ratio, *mGPS* modified Glasgow Prognostic Score, *NLR* Neutrophil-to-Lymphocyte Ratio, *PLR* Platelet-to-Lymphocyte-Ratio

In our study cohort, NLR values could be evaluated for 311/340 (92%) radiosurgically treated BM patients. Patients with NLR < 5 had a significantly longer estimated median survival after GKRS1 than those with NLR ≥ 5 (p < 0.001; Fig. 2A). For NLR groups, the univariate Cox regression model revealed a HR of 1.880 (95% CI = 1.436–2.462; p < 0.001). The multivariate Cox regression model, including sex, KPS groups, age groups, ECM status and the presence of neurological symptoms, presented a KPS < 80% (p < 0.001), age > 65a (p < 0.017), and NLR ≥ 5 (HR: 1.805; 95% CI = 1.374–2.371; p < 0.001) as independent prognostic factors for an increased risk of death.

For the continuous NLR values, the univariate Cox regression model displayed that each increase in the NLR of 1 equaled an increase of 7.2% in risk of death (HR: 1.072; 95% CI = 1.048–1.097; p < 0.001). Moreover, the multivariate Cox regression model revealed KPS < 80% (p < 0.001), age > 65a (p = 0.024), but also rising NLR values (HR: 1.060; 95% CI = 1.036–1.085; p < 0.001) as independent prognostic factors for an increased risk of death.

For the outcome evaluation in relation to IT or TT, NLR was investigated separately for patients with and without IT or TT (IT or TT treatment group). Interestingly, pre-radiosurgical NLR values < 5 were associated with a significantly longer estimated median survival in patients with IT or TT (p = 0.003), but also in patients without IT or TT (p = 0.002). Thus, in a next step, we included IT and TT groups in our multivariate Cox regression model. Even after adjusting for sex, age, KPS, ECM status, presence of neurological symptoms and IT or TT group, NLR groups remained a statistically significant independent prognostic factor for an increased risk of death (HR: 1.822; 95% CI = 1.384–2.399; p < 0.001).

### Platelet-to-lymphocyte ratio

The PLR values could be evaluated for 311/340 (92%) radiosurgically treated BM patients. Interestingly, the estimated median survival did not show any significant differences between patients with PLR < 180 and patients with PLR ≥ 180 (p = 0.226; Fig. 2B).

### Lymphocyte-to-monocyte ratio

The LMR values could be evaluated in 302/340 (89%) radiosurgically treated BM patients. The estimated median survival was significantly longer in patients with LMR ≥ 4 than in patients with LMR < 4 (p = 0.001; Fig. 2C). Consequently, the univariate Cox regression model for LMR groups displayed a HR of 1.694 (95% CI = 1.249–2.299; p = 0.001). The multivariate Cox regression model, including sex, KPS groups, age groups, ECM status and the presence of neurological symptoms, revealed KPS < 80% (p < 0.001), and LMR < 4 (HR: 1.593; 95% CI = 1.171–2.166; p = 0.003) as independent prognostic factors for an increased risk of death. 

However, the univariate and multivariate Cox regression analyses did not reveal continuous LMR values as a predictor for survival.

Furthermore, pre-radiosurgical LMR values ≥ 4 were associated with a significantly longer median survival in patients without IT or TT (p = 0.001). Nevertheless, this association could not be observed for patients with IT or TT (p = 0.195). In a next step, we included IT and TT groups in our multivariate Cox regression model. Even after adjusting for sex, age, KPS, ECM status, presence of neurological symptoms and IT or TT group, LMR groups remained a statistically significant independent prognostic factor for an increased risk of death (HR: 1.571; 95% CI = 1.152–2.143; p = 0.004).

### Modified glasgow prognostic score

Due to missing albumin values, mGPS could only be evaluated in 105/340 (31%) radiosurgically treated BM patients. From these patients, the mGPS score was 0 for 76/105 (72%) patients, 1 for 11/105 (11%) patients and 2 for 18/105 (17%) patients. The estimated median survival after GKRS1 was significantly longer in patients with a mGPS score of 0 than in patients with mGPS score of 1 or 2 (p < 0.001; Fig. 2D).

The univariate Cox regression model revealed that each increase in the mGPS score was associated with an increased risk of death (HR: 1.954; 95% CI = 1.460–2.613; p < 0.001).

Furthermore, the multivariate Cox regression model, including sex, KPS groups, age groups, the presence of ECM and neurological symptoms, showed higher mGPS groups (HR: 1.933; 95% CI = 1.447–2.583; p < 0.001), and neurologically symptomatic BM patients (HR: 1.767; 95% CI = 1.057–2.951; p = 0.030) as independent prognostic factors for an increased risk of death.

Despite the small sample size, the correlation between shorter estimated median survival after GKRS1 and increasing mGPS could be observed for patients with IT or TT (n = 55/105, 52%; p < 0.001), but also for patients without IT or TT (n = 48/105, 46%; p = 0.033). Two of 105 patients (2%) were excluded from this analysis due to missing data on IT or TT. Furthermore, in a next step, we included IT and TT groups in our multivariate Cox regression model. Even after adjusting for sex, age, KPS, ECM status, presence of neurological symptoms and IT or TT group, mGPS groups remained a statistically significant independent prognostic factor for an increased risk of death (HR: 1.836; 95% CI = 1.361–2.476; p < 0.001).

## Discussion

Despite the improvements in the management of cancer patients, the prognosis of patients with BM remains poor as it is still associated with short survival duration [17]. As the outcome of BM patients is known to be influenced by a variety of patient- but also tumor-relevant factors, the individual assessment for the optimal management with simple methods is crucial [2, 18].

In this retrospective analysis, simple laboratory parameters and their ratios, which are obtained in clinical routine, were analyzed for their prognostic values in a clearly defined cohort of Gamma Knife radiosurgically treated BM patients from different non-pulmonary PT origins (melanoma, breast cancer, GI and GU cancers).

In general, the main treatment goal for patients with BM is the local control of the metastatic lesion, in order to maintain a satisfactory quality of life while preventing death from neurological complications [17]. For local control, stereotactic radiosurgery represents a non-invasive, highly-effective method, especially for patients with multiple or deeply seated, and thereby non-resectable BM [2, 17]. Nevertheless, the therapeutic decision making for patients with multiple BM still remains challenging.

In the past, whole brain radiation therapy (WBRT) was considered to be the treatment of choice for patients with multiple BM. However, the lack of survival benefit together with the impairment in quality of life and the neuro-cognitive deterioration have recently heavily challenged the therapeutic decision for WBRT [17–19]. At our institution, stereotactic radiosurgery with a reduced prescription dose for a high number of BM is regularly performed; this procedure has also been described as an innovative therapeutic approach by other groups [17, 18].

Nevertheless, the decision of the therapeutic management should be made individually, depending on the clinical condition and prognosis of the patient [18]. Thus, easily available predictive scores are of a high interest in clinical routine and for clinical trial inclusion [7, 20].

Systemic inflammation was identified to be associated with cancer development, metastasis and progression. Similarly, markers of inflammation, such as CRP, albumin or leucocyte-based ratios, were shown to predict tumor progression in a variety of solid tumors [6, 8, 13, 14, 21, 22]. So far, data on radiosurgically treated BM patients remain scarce [5, 8]. Since NSCLC represents the most common cause of BMs, we assessed the prognostic impact of these leucocyte-based ratios in NSCLC patients with radiosurgically treated BM in a previous report [7]. The current study adds to the growing body of research on the clinical relevance of systemic inflammation-based scores, mGPS and leucocyte-based ratios, in radiosurgically treated BM patients with non-pulmonary PT, including melanoma, breast, GI and GU cancers. In this study cohort, pre-radiosurgical NLR and LMR groups were generally associated with longer survival duration after GKRS1. Of note, multivariate Cox regression models, including sex, KPS, age, the presence of ECM and neurological symptoms, revealed NLR and LMR groups as independent prognostic factors for an increased risk of death of 80.5% and 59.3%, respectively.

In addition to NLR and LMR groups, the prognostic values of the continuous metric values were also evaluated. After univariate and multivariate Cox regression models, only continuous rising NLR values were revealed as an independent predictor for an increased risk of death. Even after adjusting for age, sex, KPS and presence of neurological symptoms or ECM, each increase in the NLR of one equaled an increase of 6.0% in risk of death. A predictive value of PLR could not be observed, neither for PLR groups nor continuous PLR values.

In recent years, the advent of IT and TT has significantly changed the survival and the management of oncological patients and even of patients with multiple BM [17, 18, 23]. This benefit of IT or TT treatment was reflected in our study population as well. Patients with IT or TT showed a significantly longer survival after radiosurgical treatment than patients without IT or TT.

Thus, we additionally investigated these leucocyte-based scores for their prognostic significance in BM patients with and without systemic IT or TT at the time of first radiosurgical treatment [15, 23]. Of note, NLR and LMR groups remained statistically significant independent prognostic factors, even after adjusting for IT or TT treatment. We have previously reported that the prognostic role of NLR is superior to PLR or LMR superior in radiosurgically treated NSCLC BM patients with concomitant IT or TT [24]. The current study confirms that NLR may represent the most important leucocyte-based ratio for radiosurgically treated BM patients. Therefore, NLR could be easily applied in clinical routine to predict clinical outcome after radiosurgical treatment, even in patients with IT or TT treatment.

In addition to the leucocyte-based ratios, the pre-radiosurgical modified Glasgow Prognostic Score represented a strong prognostic predictor for survival after GKRS1 in our radiosurgically treated BM patients. Further sub-analysis for mGPS showed that each increase in a mGPS score was highly associated with an increased risk of death, even after adjusting for sex, KPS, age, ECM status, presence of neurological symptoms and IT or TT treatment. These findings were achieved despite different PT origins and limited patient numbers in each mGPS groups.

Several potential clinical parameters, such as female sex, younger age, higher KPS, and neurological asymptomatic patients, were shown to be associated with improved survival in BM patients in the previous literature and our study population [2, 25, 26]. However, pre-radiosurgical mGPS and NLR groups remained statistically significant predictors despite these potential confounders. Thus, summarizing previously published and present data, pre-radiosurgical mGPS and NLR groups seem to be the most effective and simple independent prognostic factors to predict clinical outcome in radiosurgically treated BM patients.

As we and others have commented before, the ability to predict survival in cancer patients with simple and widely available prognostic scores is of clinical importance and may help to facilitate clinical decision making and appropriate stratification of future clinical trials [7, 20, 24]. Still, we would like to further comment, that, at our institution, patients in a palliative setting or with a high number of BM are regularly treated radiosurgically if any benefit from the treatment might be anticipated. Indeed, we do not want to imply that patients with unfavorable scores should not be treated. The clinical decision should always be made according to the patients’ wishes and in the interdisciplinary agreement of the radiosurgeon and the oncologist. However, simple oncological scores and ratios may help the clinician to appropriately counsel their patients but should not prevent treatment a priori.

## Limitations

One of our study limitations was the retrospective design in a non-randomized study cohort. Furthermore, several patients had to be excluded from the mGPS analysis due to missing pre-radiosurgical albumin values. This is explained by the fact that, so far, albumin was not considered to be clinically relevant for the radiosurgical planning MRI or treatment per se.

## Data Availability

Research data will not be shared.

## Abbreviations

BM: Brain metastases
CI: Confidence interval
CRP: C-reactive protein
ECM: Extracranial metastases
fRT: Fractionated radiotherapy
FU: Follow-up
GKRS: Gamma Knife radiosurgery
GKRS1: First Gamma Knife radiosurgical treatment
GI: Gastrointestinal
GU: Genitourinary
GPA: Graded Prognostic Assessment
HR: Hazard ratio
IT: Immunotherapy
KPS: Karnofsky Performance Status Scale
LMR: Lymphocyte-to-Monocyte Ratio
mGPS: Modified Glasgow Prognostic Score
MRI: Magnetic resonance imaging
NLR: Neutrophil-to-Lymphocyte Ratio
NSCLC: Non-small cell lung cancer
PLR: Platelet-to-Lymphocyte Ratio
RPA: Recursive Partitioning Analysis
SIR: Score Index for Radiosurgery
TT: Targeted therapy
WBRT: Whole brain radiation therapy
